# Urban flooding: Coping with Weija Dam spillage by downstream communities in Ghana

**DOI:** 10.4102/jamba.v16i1.1476

**Published:** 2024-03-06

**Authors:** Gloria Asare, Michael Tuffour

**Affiliations:** 1 Faculty of Development Studies, Presbyterian University, Akropong, Ghana; 2 School of Sustainable Development, University of Environment and Sustainable Development, Somanya, Ghana

**Keywords:** dam, spillage, flooding, urban, communities, coping

## Abstract

This study adds to knowledge by specifically focusing on the dam spillage-induced floods within a fast-urbanising city with weak law enforcement in a developing country. Employing a sequential mixed-method approach, we first selected and analysed data from 120 respondents through a survey and complemented it with key informants residing in the communities affected by flooding. The findings revealed that dramatic ramifications of dam spillage-induced floods disrupt the livelihoods of many individuals in the form of disruption to income-generating activities and substantial property loss. Notwithstanding the devastating effect of the dam spillage, residents lack advance and adequate preparedness. In coping with such challenges, most flood victims rely on support from nongovernmental sources. The challenge of flooding from the dam spillage is multifaceted and deeply rooted in complex factors which includes growing urbanisation, poor urban planning and weak law enforcement. Residents’ adaptive strategies remain limited, demonstrating a dearth of resilience against such shocks.

**Contribution:**

In light of these challenges, the authors advocate a close collaborative working partnerships among stakeholders to provide flood risk management interventions, strategic planned dam spillage that minimises the effect of dam spillage-induced floods on the local communities, early warning systems and planning and enforcement of building regulations.

## Introduction

Flood disasters rank as the most common natural disaster affecting many countries across the globe (Centre for Research on the Epidemiology of Disasters [CRED] 2020; Ogie et al. 2018). Over 250 million people across the globe are affected by floods annually, with many losing their livelihoods and sometimes their lives (OECD 2016). The social, economic and environmental impacts of floods can be devastating for any country (Mensah & Ahadzie 2020). Astonishingly, the annual global cost associated with floods alone is more than $40 billion, with Asia being the most affected continent (OECD 2016; CRED 2020). In Africa, millions of people are affected by floods due to the region’s poor and limited infrastructure, high level of vulnerability of its citizens due to poverty, low adaptive capacity to flooding and limited coordination in flood management activities (Adelekan et al. 2015).

Within Ghana, the economic effect of flooding poses a major threat to the nation’s development. Countless people have endured displacement and the destruction of vast farmlands of both flood from heavy rainfalls and spillage-induced flooding. In the northern part of Ghana, for example, the Bagre Dam displaced about 100 000 people and destroyed several hectares of land in 2018. This seriously affected the livelihoods of many farmers (eds. Adegoke et al. 2019; Almoradie et al. 2020; Asumadu-Sarkodie et al. 2015a; Global Facility for Disaster Reduction and Recover (GFDRR) 2019; Musah et al. 2013; Rain et al. 2011). Among the suburb persistently buttered by floods is the Weija Township in the Greater Accra Region. The World Bank (2019) asserts that about $3.2bn worth of economic assets, including those in the Weija area, was at risk of flooding in the Greater Accra Region. One major contributor of this perennial flooding is the discharge from the Weija Dam, which has often led to loss of multi-million dollar worth of asset and loss of life (International Federation of Red Cross and Red Crescent Societies [IFRC] 2018). This flooding problem is compounded by rapid population growth in the dam vicinity, resulting in an uncontrolled urban sprawl downstream of the dam (Gyasi et al. 2018).

Several studies have focused on the causes of floods in the Greater Accra Region and how to address the challenges using exploratory literature review methodology (Abeka et al. 2020; Ahadzie & Proverbs 2011; Asiedu 2020; Asumadu-Sarkodie et al. 2015b). Nonetheless, these studies have not adequately addressed the consequences of floods on the inhabitants of the Weija municipality, which suggests that there are gaps in the literature and hence this study. Moreover, most of these studies relied on secondary data sources, drawing on a comprehensive literature review (Mensah & Ahadzie 2020). Owusu-Ansah et al. (2019) studied the general causes of the flooding within the Weija community but did not consider flooding caused by the intentional dam spillage or how individuals cope with such incidents. However, Danso and Addo (2017) emphasised the importance of understanding the coping mechanisms of flood victims in Ghana. Echendu (2022) interrogated the flood situation in Ghana and Nigeria, showing the need to partner in flood risk reduction.

This study is quite unique from the several studies in several forms. The study strictly focuses on dam-induced flooding and not rainfall. Besides, the study area is within an urban space which is fast urbanising, which has caused high land scarcity and excessive high cost of land (Appiah et al. 2014; Owusu 2008, 2013; Tuffour 2023). The study area is saddled with weak law enforcement by city authorities and has made it extremely difficult or almost impossible to evacuate residents from areas affected by floods (Allen, Apsan Frediani & Wood-Hill 2014; Owusu 2013). Moreover, most of these investigations relied on secondary data sources, drawing on a comprehensive literature review (Mensah & Ahadzie 2020), but this study uses strictly primary data. As such, this study attempts to bridge the gap in literature by employing primary data to understand preparedness for the floods, the impacts of flooding from dam spillage on the residents of Weija, as well as the coping strategies they employ to navigate these circumstances.

## Dams, flood risk and management

### Dams and floods

Dams are multifunctional structures constructed for reasons such as generating electricity, supplying portable drinking water for cities, and for irrigation to aid food production (Branche 2017). While such structures offer multiple benefits at the same time, they also carry the risk to expose downstream communities to flooding, as with the case of the Weija Township (Owusu-Ansah, Dery & Amoako 2019). The World Wildlife International (WWF) highlights that many catastrophic floods induce spillage, and even dams designed to mitigate flooding can sometimes worsen the problem (WWF 2001). Spillage is crucial to prevent dam collapse when its capacity has been exceeded in an event of heavy rainfall. However, poorly designed dams, inadequate spillways, excessive water released and untimely emergency release are some factors that exacerbate flooding downstream (Lempérière 2017; WWF 2001).

Dam spillage poses substantial socioeconomic problems over extended periods, including economic strain of rebuilding lives, properties and communities (IFRC 2018). In Ghana, the Bagre Dam spillage in Burkina Faso has raised significant concerns and has been blamed for the frequent flooding in parts of Upper East Region due to the increased water discharges (Adegoke et al. 2019; Almoradie et al. 2020). Such extreme flooding from dams undermines their intended purpose, which includes reducing downstream flooding and meeting the needs of communities and floodplain farmers who rely on the river for their livelihoods (Gujja 2006; WWF 2001).

Noteworthy examples of dams causing flooding extend beyond Ghana and Burkina Faso. For instance, the catastrophic 2011 Brisbane floods in Australia were influenced by climatic factors and water released from the Wivenhoe Dam (Van den Honert & McAneney 2011). This event affected over 200 000 people, with insurance claim reaching $2.5bn (Van den Honert & McAneney 2011). Similarly, an investigation of upstream structures on downstream in North and South Korea concluded that dam water release contributed to flooding (Ha, Kim and Bae 2020). In West Africa, rainfall and spillage from the Bagre Dam trigger flooding in Burkina Faso and Ghana, raising concerns of potential transboundary conflict (Adegoke et al. 2019; Almoradie et al. 2020). In 2018, the dam caused unprecedented floods in two regions in northern Ghana, affecting about 32 000 individuals and resulting in nearly 10 fatalities (IFRC 2018). These floods have not only affected people and infrastructure but have also disrupted essential livelihood activities (Mensah & Ahadzie 2020).

Beyond dams, several other factors contribute to the increasing risk of urban flooding (Price & Vojinovic 2008). Climate change is a major driver, with more frequent and extreme rainfall patterns expected to increase the severity of urban flooding, potentially worsening dam spillages (IPPC 2007; Miller & Hutchins 2017). Urbanisation is another major problem, as green lands and vegetation have diminished at the expense of new developments to accommodate the increasing population of people migrating to towns and cities for better lives (Handayani et al. 2020; Rahman et al. 2021; Suriya & Mudgal 2012). Flood-prone areas are now settlements, and this is limiting the natural water storage for floodwaters, thereby increasing the risk of flooding (Suriya & Mudgal 2012). Unplanned development around flood plains increases the vulnerability to flooding, therefore a threat to achieving sustainable flood management.

### Flood management

Flood risk encompasses three key components: the presence of a hazard, the vulnerability of the system, and the the exposure to risk (Birkmann 2013; Crichton 1999). Flood risk management (FRM) aims at reducing the probability of floods or the consequences of floods when they occur. This pursuit involves the deployment of structural (physical) and nonstructural measures (Gersonius et al. 2016; Jha, Bloch & Lamond 2012; Price & Vojinovic 2008; Rose et al. 2016; Shah, Rahman & Chowdhury 2018). The structural strategies involve the use of physical interventions (e.g. flood barrier, defence, levee), which creates a barrier between the source of risk and vulnerable areas (Gersonius et al. 2016; Jha et al. 2012; Penning-Rowsell & Peerbolte 1994). Though effective in flood prevention, these measures are not always applicable and are often costly investments as well (Owusu, Wright & Arthur 2015; Sayers et al. 2013). Nonstructural measures, such as floodproofing, preparedness, flood warning and recovery, help to reduce the impact of floods when they occur especially at the property level (Owusu, Wright & Arthur 2015; Rose et al. 2016). Both strategies enhance the flood resilience of communities (Restemeyer, Woltjer & Van den Brink 2015).

In the context of Africa, and Ghana, huge investment is needed in flood-resilient infrastructures to adapt to the frequent flooding. Achieving sustainable flood management requires collaboration among all FRM stakeholders. This is essential especially in Africa, as fragmented approaches and poor implementation have hindered effective flood management (Adelekan et al. 2015). In reporting on Ghana’s FRM efforts, Almeradie et al. (2020) highlighted some challenges such as the reactive approach to flood management and poor implementation and monitoring of flood early warning systems (FEWS). These problems call for more inclusive community participation in FRM policies and action plans, where central governments must play a leading role in funding and implementing large-scale measures to reduce flooding and protect people (Almeradie et al. 2020). In the case of flooding from the Weija Dam, stakeholders including the Ghana Water Company, Land Use and Spatial Planning Authority and Community leaders have essential roles in addressing the problem. Moreover, one other challenge with public FRM institutions in Africa is their low capacity which hinders effectiveness. In particular, the National Disaster Management Organisation (NADMO) of Ghana, which is at the forefront in handling all forms of disaster including floods, is often criticised in its operations due to its several limitations including weak coordination of FRM activities (Almeradie et al. 2020). This important institution needs help to build capacity to provide adequate relief and recovery support for flood victims.

While relocating flood vulnerable to ground might seem a logical solution, it is not always feasible due to cost and livelihood implications (Douglas et al. 2008). Financial constraints, lack of resources and alternatives hinder evacuation efforts, leaving residents to the devastating effects of flooding (Douglas et al. 2008; Jha et al. 2012; Okaka & Odhiambo 2019; Owusu-Ansah et al. 2019). Therefore, a combination of nonstructural and structural adaptive measures becomes essential in flood-vulnerable communities to mitigate flood impacts (Owusu et al. 2015; Rose et al. 2016). Flood risk awareness, early flood warning and effective flood communications are pivotal for flood preparedness. Coping strategies such as structural adjustment, sandbags and drainage construction are some measures individuals undertake to minimise the effect of flooding (Owusu-Ansah et al. 2019).

## Research methods and design

### Study area

**FIGURE 1:**
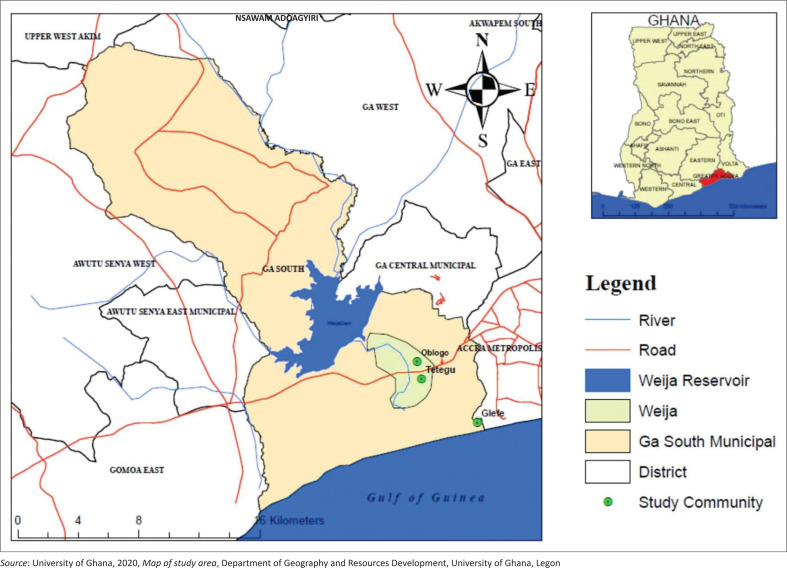
Map of the study area.

The study was conducted in Weija, situated within the Ga South Municipal of the Greater Accra Region of Ghana. The municipality is located within latitudes 5°47’30” N and 5°27’30” N and longitudes 0°31’30” W and 0°16’30” W and lies in the dry equatorial climatic zone with two rainfall seasons (Figure 1). Annual mean rainfall varies between 790 mm and 1270 mm, while average temperatures range between 25.1 °C in August and 28.4 °C in February and March (Ghana Statistical Service [GSS] 2014). A major landmark within the municipality is the Densu River, originating from the Atewa Range near Kibi and flowing a distance of 116 km into the Weija Reservoir. The Weija Dam was constructed in 1978 with the purpose of supplying water across 20 km to residents of the Greater Accra Metropolitan Area. Managed by the Ghana Water Company Limited, the dam supplies about 80% of potable water for Accra and its surroundings. The dam has an area of 20.5 km^2^, a maximum normal water level of 15.25 m, and a maximum storage capacity of 143.11 × 10^6^ m^3^ (Kuma & Ashley 2008) of its establishment and proximity to Accra’s central business district has transformed Weija from a remote rural area to an urban settlement.

The dominant economic activities in the area are fishing, farming and trading. In 2010, the population was recorded at 16 000, showing an average growth rate of 12% (GSS 2014). Weija has increasingly become vulnerable to flooding, with many communities and properties at high risk of flooding (Boateng 2021; Owusu-Ansah 2019). The flooding in the Weija Township is associated with both rainfall and spillage of the dam, which affects many downstream communities. The communities selected for the study are Tetegu, Oblogo and Glefe communities in the Weija Township (see Figure 1), which fall within the medium- to high-risk flood zones (Owusu-Ansah 2019). These communities have consistently been inundated by floods from the dam spillage and featured in news headlines and discussions about flood disasters in Weija. Therefore, they offer a good representation of areas prone to flooding.

### Data collection and analysis

The study employed a sequential mixed-method approach, with a dominant focus on quantitative methodology, supported by the qualitative insights (Bryman 2012; Creswell & Clark 2017; Creswell & Creswell 2017; De Silva 2011; Jogulu & Pansiri 2011; Saunders, Lewis & Thornhill 2019). Quantitative data were gathered from households in the flood-affected communities, while qualitative data were solicited from officials of the national FRM and NADMO. The qualitative data were recorded and analysed to corroborate the quantitative findings. The survey questions were designed to solicit responses on the causes of floods, its impact, their flood preparedness and coping strategies of residents. Even though the households had experienced periodic floods from the dam spillage, a particularly devastating flood from the same source occurred in October 2020. The study used a two-stage sampling method for the quantitative data collection. Firstly, communities were randomly selected with Tetegu, Oblogo and Glefe chosen as primary locations when the listing of the suburbs was done. Secondly, 120 households were randomly selected from the list comprising 64, 30 and 26 from Tetegu, Oblogo and Glefe, respectively. With the qualitative data, we purposively selected key informants who were very conversant with the research subject in the study area. The quantitative data were analysed using descriptive statistics. The qualitative data were subjected to thematic analysis where we created the right codes and themes (Saldana 2021) to augment the results from our quantitative analysis.

### Ethical considerations

The study complied with fundamental ethical principles in social research, which does not expose the shortcomings of respondents or communities visited or posed any form of harm to them (eds. Menéndez, Paoletti & Tomás 2013).

## Results

### Sociodemographic background of respondents

As shown in Table 1, our survey constituted a greater proportion of women (78%) compared to men (22%). This distribution is quite different from the demographic makeup at the municipality, where women constitute about 52% of the gender classes (Ga South Municipal Assembly 2021). The results also show that most of the respondents (98%) were within the economically active age bracket of 20–59 years. Age groups of 30–39 years (32%) and 40–49 years (37%) were the majority respondents. In terms of educational background, more people had completed junior high school (38%) and senior high school (20%), but 29% of the respondents had no formal education. This noticeable proportion of individuals without formal education and the small tertiary-level respondents (4%) imply that the educational status of the respondents was lower than the national and municipal statistics (GSS 2018).

**TABLE 1:** Demography of respondents.

Demographic data	Frequency	%
**Sex**		
Male	27	22.5
Female	93	77.5
**Age (in years)**		
20–29	17	14.2
30–39	39	32.5
40–49	45	37.5
50–59	17	14.2
60–69	2	1.7
**Education**		
Junior high school (JHS)	45	37.5
Senior high school, vocational or technical	24	20.0
Tertiary	5	4.2
Primary	11	9.2
No formal education	35	29.2
**Occupation**		
Trader	73	60.8
Artisan	20	16.7
Farmer	11	9.2
Fisherman	10	8.3
Others (teacher, health worker, etc.)	6	5.0
**Marital status**		
Married	68	56.7
Not married	52	43.3
**Religion**		
Christian	107	89.2
Non-Christian	13	10.8

The results reveal that most (95%) of the respondents were engaged in the informal sector, with only 5% employed in the formal sector either as teachers or health workers. Those involved in trade formed the majority (61%) of all the occupations reported, primarily dominated by women, followed by artisans (17%). The occupation distribution implies that many respondents may earn modest incomes. In addition, 57% of the respondents were married, while 43% were not married. The survey respondents were largely Christians (89.2%), with 10.8% as non-Christians belonging mainly to Islam. The results on marital status and religious affiliation were similar to the national figures (GSS 2018).

### Information-induced preparedness towards dam spillage-induced floods

Given the havoc that sudden disasters such as flooding can cause, it is expedient that people are prepared in advance to minimise its consequences. Notwithstanding the preparation, our key informants revealed that their preparations against flooding fall short. As a respondent expressed:

‘Some people may have some measures like constructing drains in front of their property to reduce the floods. But these are small drains as the floods are huge and so may not be effective. People cannot do much because their buildings are already in the floodplains.’ (Key Informant Interview, November 2020)

In view of this, we asked residents about their sources of flood information prior to flooding. It was not surprising that about 54% of the respondents said they relied on the media (radio) for information on flooding and impending Weija Dam spillage (see Table 2). Nearly 30% of the respondents received information from NADMO. A further 15% depended on the community leaders, assemblymen and others (including personal interactions) for information about dam spillage and impending floods.

**TABLE 2:** Sources of information on flood preparedness.

Sources of information	Frequency	%
NADMO	37	30.8
Assemblyman	4	3.3
Community leaders	9	7.5
Media (radio news)	65	54.2
Others	5	4.2

**Total**	**120**	**100**

NADMO, National Disaster Management Organisation.

The flood information or alert regarding dam spillage gets to the residents too late, primarily via radio medium or through interaction with neighbours. Consequently, many people are unable to prepare adequately and in advance. In some cases, people do not receive any information at all making them face dire consequences.

### Impact of dam spillage-induced flooding on residents

The insights gathered from respondents underscore recurring flood damage to critical infrastructure including essential services. The effect is pronounced on services such as clean and safe water, electricity, transport, communication, education and healthcare services. A total of 10% of respondents reported the loss of a family member in the most recent dam-related spillage, which is a grievous setback for the affected communities. The livelihood activities of residents were affected by the dam-induced floods, as most of them were actively engaged in the informal sector. Artisans and traders are unable to work regularly during the duration of the floods which sometimes continue up to 1 month, disrupting their income-generation activities. Women traders and artisans who constituted 90% and earn daily wage are mostly affected by floods. Also, agricultural activities are affected, as farmlands and crops are submerged or completely swept away by floodwaters. Overall, floods disrupt the economic activities of residents and lead to loss of livelihoods in the communities. From Table 3, the study reveals that personal properties of 58% of the respondents were damaged through floods. These include houses and household items such as mattress, television, livestock fridge, cell phone and furniture.

**TABLE 3:** Property mostly loss through floods from the dam spillage.

Variable	Frequency	%
**Loss of property by flood**		
Yes	97	80.8
No	23	19.2
**Type of property damaged**		
House building and/or structure damage	17	17.5
Household items, carpets and/or floor	11	11.3
Mattress and/or bedroom items	13	13.4
Television	11	11.3
Fridge and/or freezer	6	6.2
Food	5	5.2
Cell phone	3	3.1
Furniture	15	15.5
Domestic animals livestock, pets and others	10	10.3
Farmland produce and crops	6	6.2

The interviews stressed the major sufferings of residents from flooding caused by dam spillage. The loss of lives from the dam spillage is often due to the depth and velocity (fast-flowing) of the floodwaters which is so dangerous and can sweep away any object on its path. Roads and drainages are often inundated and, at the household level, people can lose their possessions and incur significant structural damage. The livelihood of people is affected throughout the duration of the floodwaters. The impact is harsh on artisans and traders whose workplaces are right within the flood-prone areas as they are disconnected from their businesses, with no income.

The majority of the respondents (75%) indicated that flooding normally lasts between 1 and 2 weeks after the dam spillage. While 23% stated that flooding could last for 3 weeks to 1 month, the remaining 1.7% indicated that floods can last longer than 4 weeks. Given the flood durations in Table 4, flooding could impact economic activities in Weija and impede access to services such as health, education and electricity over an extended period, causing further troubles.

**TABLE 4:** Flood duration of dam spillage-induced flood.

Duration of flood	Frequency	%
One week	41	34.2
Two weeks	49	40.8
Three weeks	19	15.8
Four weeks	9	7.5
More than 4 weeks	2	1.7

**Total**	**120**	**100**

The interviews with officials confirmed the seriousness of flooding within the study area, primarily instigated by rainfall-induced dam spillage. They underscored the severity of these flood incidents, particularly in terms of the extent of inundations and depth of floodwaters. It was revealed that the sheer volume of water released at a given time can cause devastating consequences, with the dam spillage occurring either once or twice annually depending on the rainfall patterns within the study area and its environs.

### Post-flood coping mechanisms

**FIGURE 2:**
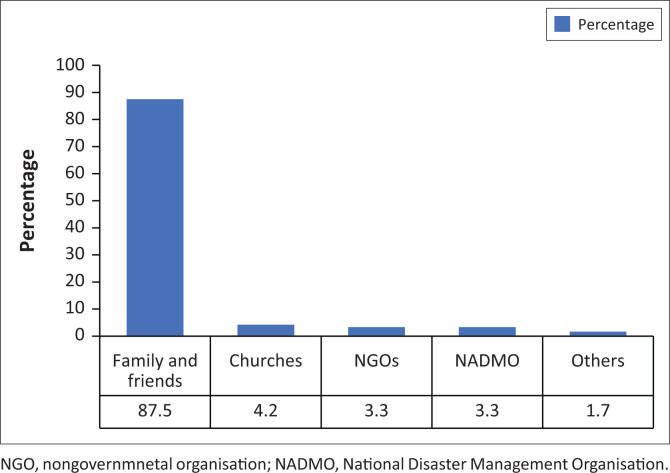
First and most reliable source of support during floods.

The study showed that the family support systems and social networks were pivotal in helping flood-affected people to cope and recover from flood events. A noteworthy 88% of the respondents affirmed their reliance on family and friends for support in the aftermath of a dam spillage flood incident (see Figure 2).

An equal 3.3% each noted that they relied on NADMO and NGOs for support. About 4% of the respondents indicated that their main source of support was from churches. NADMO, Ghana’s principal institution responsible for flood-related issues, played a critical role in offering relief assistance to flood victims, including life jackets, blankets and mattresses. However, more than 70% of the respondents expressed the sentiment that the services provided by NADMO fall short, thereby calling for greater efforts in helping flood victims. In terms of coping mechanisms, some residents have made structural adjustments to their buildings, drainage systems and property floors to reduce the damage from floodwaters. This finding is similar to the observations by Owusu-Ansah et al. (2019), which highlighted strategies such as the use of stones and sandbags, strengthening of walls, and the use of water pumps as coping mechanisms for households.

### Flood mitigation and management

Table 5 depicts the responses when respondents were asked about their main approach or preference for mitigating flooding in the communities. A significant 87.5% expressed the belief that the dam spillage and subsequent flooding can be reduced by regularly desilting the dam to maintain its water storage capacity. A smaller proportion advocated for the relocation of the affected people to higher elevations (9.2%), while only a few people recommended that farming along the banks of the Weija River should stop (3.3%). The inclination towards desilting aligns with an earlier study that reported a decrease in the dam’s total volume due to siltation caused by farming activities and new settlements around the dam (Kuma & Ashley 2008).

**TABLE 5:** Key strategies of reducing impact on flooding by dam spillage.

Strategies	Frequency	%
Desilting of dam	105	87.5
Move to higher grounds	11	9.2
Stop farming on river banks	4	3.3

**Total**	**120**	**100**

Further perspectives on flood management strategies are reported in Table 6. A notable 82% of respondents endorsed early warning systems as the best option of managing floods arising from dam spillage. Additionally, 10% viewed early sensitisation of flooding as the best option, while 8.3% perceived that developing alternate coping mechanisms would help build resilience and adaptation.

**TABLE 6:** Views on managing floods from Weija Dam spillage.

Management of the spillage	Frequency	%
Early warning systems	98	81.7
Early flood sensitisation	12	10.0
Alternative coping mechanism	10	8.3.0

**Total**	**118**	**100**

The findings from the interview with the NADMO official underscored the problem of new developments in unauthorised areas around the dam. This uncontrolled construction in flood plains is impeding the natural waterways, exacerbating flooding occurrences. Such acts have necessitated demolishing of buildings in close proximity to the dam and river body in an attempt to reduce flood incidence. The interaction revealed that the local authorities are engaging more with the communities and traditional chiefs on the pressing issue of flooding. They indicated an ongoing flood education to help the communities cope with the dam spillage-induced floods. However, the officials stressed the need for government support to undertake some local-level flood mitigation initiatives. They revealed a comprehensive plan for a major drainage construction to help reduce flooding, though it may take time to materialise. NADMO also iterated the government’s plan to deal with the flooding problem in the Weija Township, including the need for a new drainage system. While this is a long-term solution, the present actions by NADMO and other authorities to reduce flooding include regular dredging and desilting of main drains within the municipality. These are geared towards ensuring that the drains and dam perform at their optimal capacity.

## Discussion

The findings of the study indicate that most of the surveyed residents are engaged in informal livelihood activities, such as trading, farming and fishing downstream of the Weija Dam. These individuals are highly vulnerable to flooding, with their sources of income susceptible to being inundated by floods from the dam spillage. Planning prohibitions around the dam area has not worked, as many people have encroached on the floodplain with developments progressing year-round. This has exposed a substantial number of residents to flood risk, with the potential failure of the dam carrying serious implications. If the flooding protracts after the dam spillage, it causes a lot of discomfort that these events can cause. Notably, longer duration floods (> 12 h) are much damaging than shorter floods, and awareness of flood risk and its characteristics is crucial in implementing sustainable flood management (Penning-Rowsell et al. 2003; Shah et al. 2018). The impact of flooding from the dam spillage was noteworthy, as the inundation disrupted the economic and livelihood activities of the residents, notably trading and farming. Elsewhere, a study in northern Ghana reported the damaging effect of floods on the principal livelihood of people which is farming (Musah et al. 2013). At the residential level, 58% of respondents reported damage or loss of personal items because of floodwater entering their homes. Items that were lost or damaged, including TVs, fridges and mattresses, accounted for more than 60% of the total items enumerated. Structural damage to building accounted for about 18%. These findings align with studies in the past which highlighted the extent of flood damage at the property level, urging greater protection for vulnerable residents to reduce flood damage (Owusu et al. 2015).

In terms of personal flood mitigation measures, few residents resorted to the use of sandbags and structural adjustments to buildings as means of minimising the impact of flooding. Nonstructural flood protection measures such as Flood Early Warning and Communication are critical measures that give individuals sufficient time to prepare for floods, ultimately saving lives and reducing flood losses (Cools, Innocenti & O’Brien 2016; Kundzewicz 2013). This perspective resonated with a substantial majority (92%) of the respondents who endorsed the implementation of early warning systems as the first and best option in flood management in Weija’s flood management. In addition, a greater proportion (88%) of the respondents proposed that desilting of the Weija Dam would reduce downstream flooding. This viewpoint aligns with studies on dam or reservoir water storage, which suggest that siltation often caused by human activities, including farming, contributes to reductions of 4%–33% of the designed dam storage (Adwubi et al. 2009; Sekyi-Annan et al. 2018).

The study further examined the coping strategies adopted by flood victims and found that a significant proportion (88%) relied on friends and relations for support at the aftermath of floods. Surprising, only 3% turned to NADMO, the principal responder in such incidents. This sustaining process of flood recovery is consistent with previous findings by Danso and Addo (2017). Although flood victims received support from the state, most people (77%) were disgruntled about the services provided by NADMO and wished for better services. This observation may agree with concerns that this organisation needs enhanced coordination and decentralisation of operations to ensure more effective services and flood management (Almeradie et al. 2020).

The findings of this study open a discourse on effective flood management within the study area and similar challenges elsewhere, in particular, the construction of dam for a reason and its role in recurrent flooding episodes, in addition to addressing the question of whether we should save the dam or protect the vulnerable residents and their livelihoods. Both are of prime importance. Relocating affected settlements to low-risk areas seems a viable option of flood management. However, this proposal presents challenges and does not guarantee new developments in floodplains areas. Besides, only 4% of respondents endorse relocation and cessation of farming along river banks as the means of mitigating flooding. The majority perspectives diverge from this view, and therefore a holistic approach may be required. An integrated approach to flood management, considering factors such as climate change, urbanisation, unplanned development, among others, is necessary for urban flood management (Bubeck et al. 2017). Additionally, urban stormwaters through measures such as sustainable urban drainage systems and green infrastructure are emerging solutions in urban flood management (Price & Vojinovic 2008; Webber et al. 2020).

The Greater Accra Metropolitan Area where Weija is located is confronted with a situation where intense land scarcity is an everyday reality. Actually, there are not new lands to relocate these large communities; therefore, these residents though several have decided to stay in this danger zone of flooding and cope with the difficulty. Even though there is land, the scarcity in GAMA is significant; there should be a conscious effect to evacuate residents from the area and make sure the law works to the latter. Even though it is going to be difficult, financially, socially and culturally, it will call for stakeholder engagement involving residents, government and traditional leaders to make it successful. The communication of flood information is a useful indication, as the flood forecast itself and therefore effective relaying of warnings using appropriate media is necessary to reduce the risk and effect of flood. As communication and transportation are relatively available in the urban space, city authorities could capitalise on it to rescue residents in real quick time. Also, enhancing the adaptive capacity to flooding, especially in less developed countries, requires strengthening weak national institutions and resourcing them for effective operation (Almeradie et al. 2020). To effectively address the problem, it is important that policies aimed at tackling them, such as prohibiting unplanned development and better land use management, are strictly enforced without compromises.

While people affected by floods take personal actions to cope or survive, including making structural adjustments to property, there is a pressing need for more support to enable them adapt to frequent and severe floods. The adaptive capacity of communities can be improved through flood education and effective engagement in flood management. National FRM institutions like NADMO should be resourced and decentralised to support vulnerable communities in implementing strategies to enhance flood resilience, as well as aiding in recovery following flood incidents.

## Conclusion

The purpose of the study was to examine the impact of floods on the downstream communities of the Weija Dam and their coping strategies. Flooding can have damaging effects on residents. This includes the disruption of public infrastructure, loss of livelihood activities and loss of personal property such as housing structure and household contents. The consequences of floods on people’s livelihoods can increase poverty within flood-prone settlements, thereby undermining efforts in the global goals of eradicating poverty and hunger in the world. The economic consequences of flooding are particularly severe for any country, especially for the least-developed nations of the world.

This clearly indicates that with the urban space such as areas close to the Weija Dam, land scarcity and weak law enforcement within the study area have made it almost impossible to relocate residents from the flood zone to safer places. Preparedness to the effect of the dam spillage is not apt even though residents insist that early flood warning system is a crucial coping mechanism. The dam-induced spillage creates socioeconomic hardships for residents but very limited support comes to them.
